# Evidence for Rapid Oxidative Phosphorylation and Lactate Fermentation in Motile Human Sperm by Hyperpolarized ^13^C Magnetic Resonance Spectroscopy

**DOI:** 10.1038/s41598-017-04146-1

**Published:** 2017-06-28

**Authors:** Steven Reynolds, Nurul Fadhlina bt Ismail, Sarah J. Calvert, Allan A. Pacey, Martyn N. J. Paley

**Affiliations:** 1 Academic Unit of Radiology, Department of Immunity, Infection and Cardiovascular Disease, Medical School, University of Sheffield, Royal Hallamshire Hospital, Sheffield, S10 2JF UK; 2 Academic Unit of Reproductive & Developmental Medicine, Department of Oncology and Metabolism, Medical School, University of Sheffield, The Jessop Wing, Sheffield, S10 2SF UK

## Abstract

Poor sperm motility is a common cause of male infertility for which there are no empirical therapies. Sperm motility is powered by adenosine triphosphate but the relative importance of lactate fermentation and Oxidative Phosphorylation (OxPhos) is debated. To study the relationship between energy metabolism and sperm motility we used dissolution Dynamic Nuclear Polarization (dDNP) for the first time to show the rapid conversion of ^13^C_1_-pyruvate to lactate and bicarbonate, indicating active glycolytic and OxPhos metabolism in sperm. The magnitude of both lactate and bicarbonate signals were positively correlated with the concentration of progressively motile sperm. After controlling for sperm concentration, increased progressive sperm motility generated more pyruvate conversion to lactate and bicarbonate. The technique of dDNP allows ‘snapshots’ of sperm metabolism to be tracked over the different stages of their life. This may provide help to uncover the causes of poor sperm motility and suggest new approaches for novel treatments or therapies.

## Introduction

Poor sperm quality is a major barrier to conception and is thought to contribute to 30–50% of cases of infertility in heterosexual couples^1^. Whilst some of these problems are due to men producing inadequate numbers of sperm (oligozoospermia), or no sperm at all (azoospermia), a significant proportion will be caused by the fact that the sperm being produced are poorly motile: a diagnosis called asthenozoospermia^2^. Following coitus, sperm must navigate through a complex series of microenvironments in the female reproductive tract to reach the egg^3^, however, many men have too few motile sperm for this to occur successfully and current techniques of motility assessment (see Supporting Video V1) are unable to determine why this is the case. Energy metabolism is necessary in the generation of ATP to maintain sperm function and sustain motility^4^. As such, this paper aims to gain a better understanding of sperm energy metabolism, and its relationship with sperm motility, by the first application of dissolution Dynamic Nuclear Polarization (dDNP) to show rapid pyruvate metabolism in human sperm.

**Figure 1:**
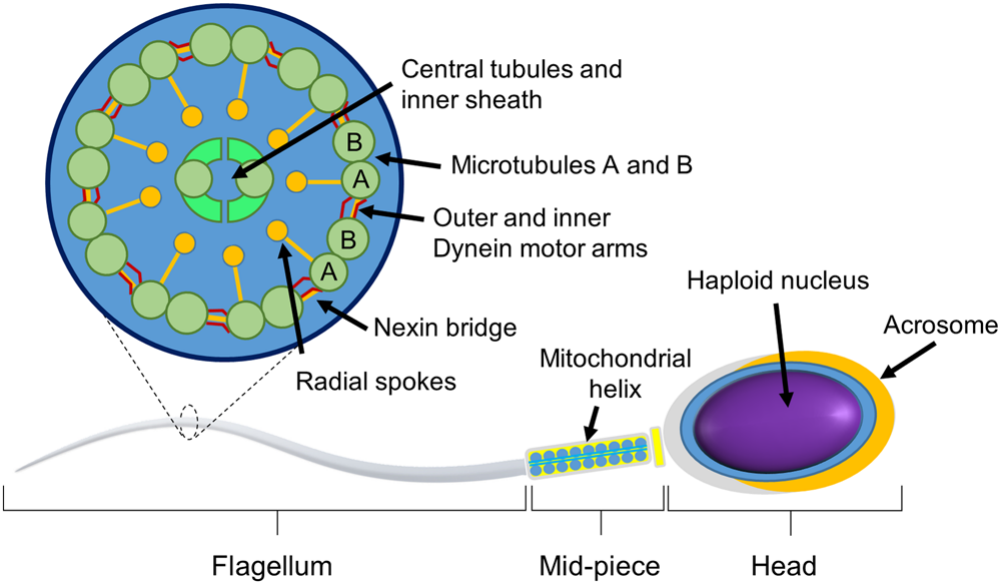
Cartoon depiction of a sperm showing: (i) the head, containing the haploid nucleus and capped with the acrosome for breakdown of the zona pellucida; (ii) mid-piece, comprised of a mitochondrial sheath; and (iii) flagellum, axoneme of the flagellum extends from the posterior of the head comprised of microtubules surrounded by a plasma membrane. Dynein motor proteins and nexin linkages span between microtubules A and B.

Sperm are highly specialized cells that deliver the male genome to the oocyte (Fig. 1). The sperm head contains densely packed and transcriptionally inactive^5^ haploid DNA, along with some RNA carried over from spermatogenesis^6, 7^ and is capped by the acrosome containing enzymes capable of digesting the zona pellucida at fertilization^8^. Posterior to the head is the flagellum that extends to 90% of the sperm’s length and contains a 9 + 2 arrangement of microtubules and motor proteins which provide the sperm with propulsive force to move^9^. Finally, proximal to the head, and surrounding the flagellum, is the mid-piece which contain the mitochondria^10^ arranged in a helical sheath with little cytoplasmic volume.

Proteomic analysis of the human sperm head and flagellum (including the mid-piece) shows that sperm are highly differentiated in terms of their protein functions, with the tail and mid-piece being dominated by enzymes associated with lactate fermentation (e.g. Lactate Dehydrogenase; LDH) and OxPhos (e.g. Pyruvate Dehydrogenase; PDH)^5^. The generation of adenosine triphosphate (ATP) is essential for sperm motility^11^ and the two main energy pathways are: (a) glycolysis within the cytosol; and (b) Oxidative Phosphorylation (OxPhos) by the mitochondria. There have been numerous studies in the literature examining the relative importance of glycolysis and OxPhos (reviewed by du Plessis^4^ and Ferramosca)^12^. However, the conclusions are confused by the use of differing species, with variations in the structural details of head and tail^13, 14^, dominance of energy pathway, the preferred substrate used for generating ATP and whether ATP diffuses from the mid-piece into the tail^4^. Furthermore, the extent to which sperm use internal stores of metabolites, or rely on an exogenous supply from the external environment is debated^15^.

To help resolve this debate, isotopically labelled substrates can be used to track a metabolic pathway depending on the location of the isotope label. For example, catalysis of ^13^C_1_-pyruvate by LDH and PDH will label lactate in the C1 position and bicarbonate respectively. Whereas the same enzymes will catalyse ^13^C_2_-pyruvate to ^13^C_2_-lactate and ^13^C_1_-acetyl-CoA but not bicarbonate. Combined with Magnetic Resonance Spectroscopy (MRS), the use of ^13^C labelled substrates allows molecules along a metabolic pathway to be, in principle, observed in the spectrum. Previously, non-human sperm has been incubated with ^13^C-glucose and the results shown that both lactate fermentation and Oxidative phosphorylation are active^16, 17^. Whereas, human sperm have been incubated with ^13^C labelled of glucose and pyruvate for two hours and metabolism to lactate detected in supernatant of methanol extracted cells^18^. Preliminary work by our group has shown that the metabolism of ^13^C labelled substrates by live human sperm can be tracked by MRS over a 20 hour period^19^. Since human sperm are able to remain viable for up to 6 days within the female reproductive tract^3^, conventional ^13^C MRS is a suitable technique for tracking long term changes in sperm metabolism. However, since aspects of sperm physiology can take place over several minutes (e.g. the onset of hyperactivated motility)^20^, a more rapid technique is required to observe any metabolic changes which may underpin them.

**Figure 2:**
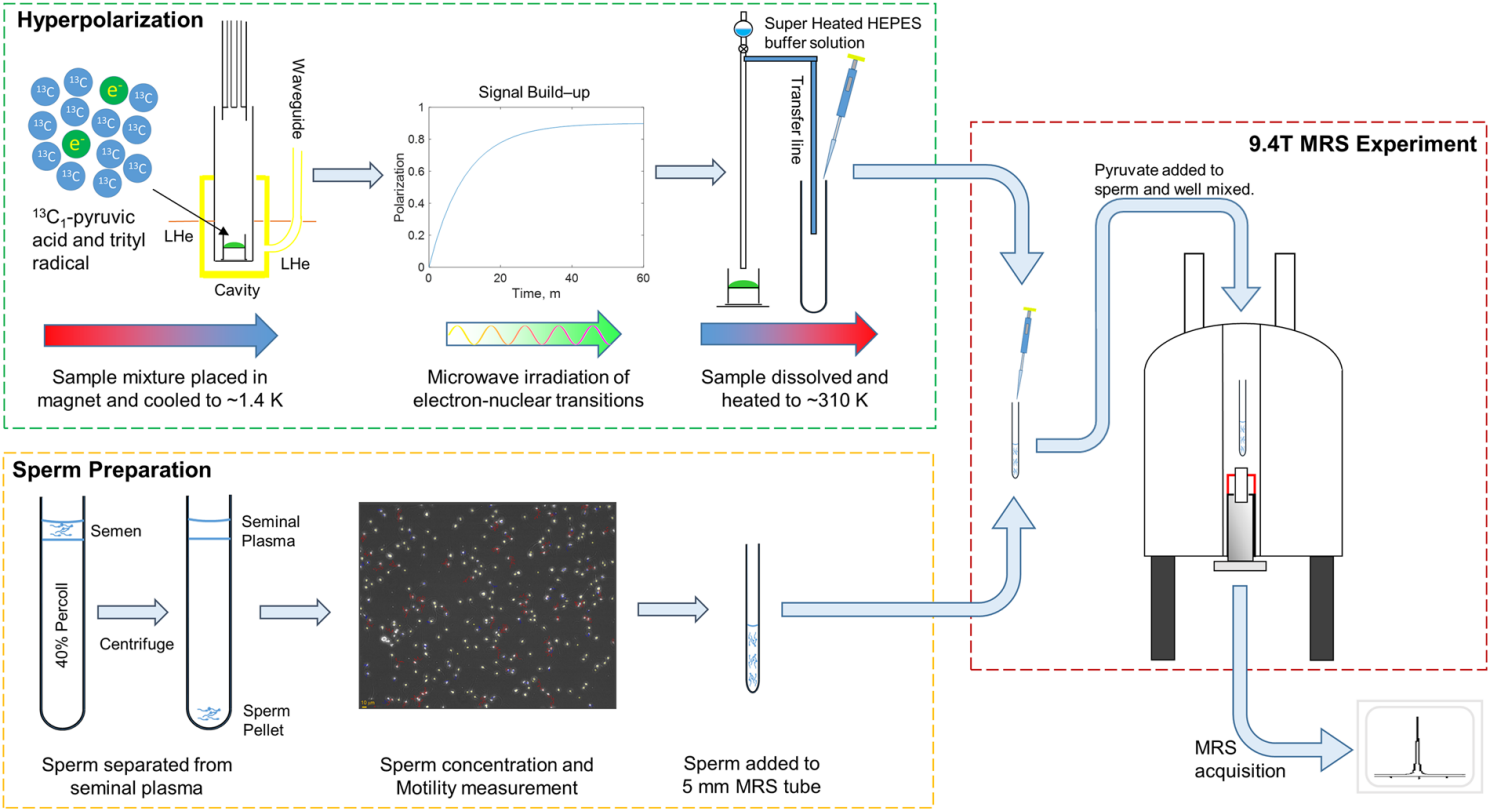
Outline of the methods used for administering hyperpolarized substrates to live sperm. The method consists of three parts, briefly: (i) Hyperpolarization, a mixture of pyruvate/trityl radical was cooled to 1.4 K and microwave for ~1 hour. Once the hyperpolarized ^13^C_1_-pyruvate signal had built up, the sample was then rapidly dissolved with superheated HEPES buffer solution and returned to ~310 K; (ii) Sperm Preparation, the semen sample was layered on top of a denser Percoll solution and centrifuged. Sperm are pelleted at the bottom, with seminal plasma remaining at the top of the tube. After further washing, sperm are assessed for concentration and motility before being placed in an MRS tube; and (iii) MRS Experiment, quickly after the pyruvate sample was dissolved and collected, an aliquot was taken and injected into the MRS tube containing sperm and well mixed. The MRS tube was placed in the magnet and MRS scanning commenced. See Methods for more details.

The technique of dDNP has emerged as a technology that increases the available MRS signal by many orders of magnitude^21^. The technique exploits the fact that at very low temperature the electron polarization of a stable free radical becomes almost 100%, and that this polarization can be transferred by microwave radiation to nearby molecules containing nuclei such as ^13^C. Once the target molecules have become hyperpolarized the sample is rapidly returned to room temperature for use in experiments (Fig. 2). Combining it with ^13^C labelled molecules allows *in vivo* monitoring of metabolism kinetics by rapidly acquiring a series of MRS spectra over a short time window, without the interfering background signals experienced in ^1^H MRS^22^. More recently the methodology has been extended into human studies of prostate cancer^23^ and heart metabolism^24^. The technique has also been used for *in vitro* detection of cellular metabolism^25^.

The enhancement provided by dDNP to acquire multiple ^13^C spectra in seconds provides the ability to trace rapid changes in sperm metabolism at different functional stages through their life after ejaculation. Here we show for the first time the relative flux of hyperpolarized ^13^C labelled pyruvate through lactate fermentation and OxPhos and their relationship to sperm motility.

## Results

**Figure 3:**
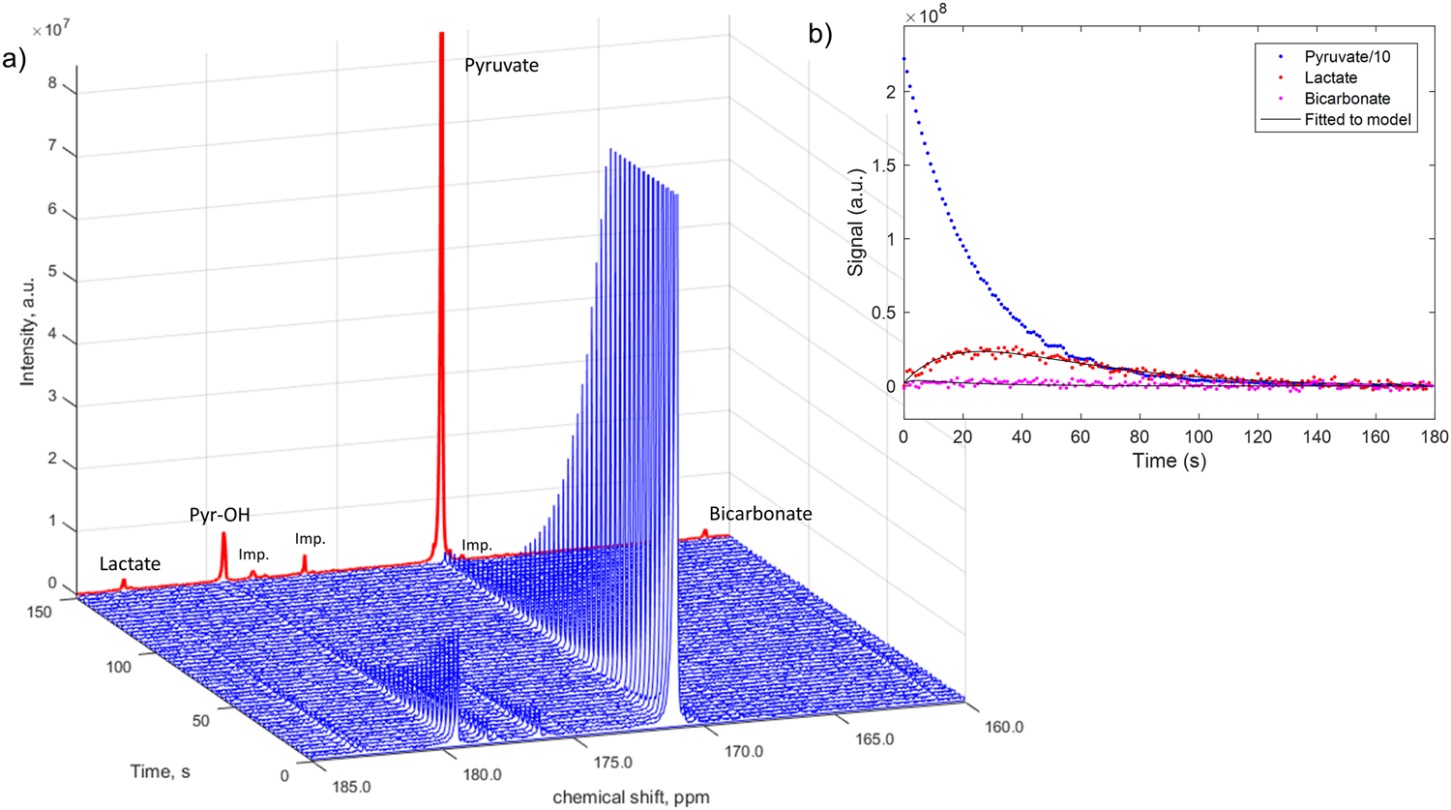
(**a**) Plot of stacked spectra (blue) acquired every 1 s over 180 s. Every 3^rd^ spectrum from 1–150 s shown for clarity. The sum of all spectra in the time course (normalized to the maximum intensity of the time course spectra) is shown in red. Other peaks observed in the hyperpolarized spectrum were: (i) pyruvate-hydrate (Pyr-OH), a commonly observed, non-metabolically active, molecule that is in fast chemical exchange with pyruvate; and (ii) other unknown impurities present in the supplied ^13^C_1_-pyruvic acid. The impurities were confirmed by their presence in the spectra of a control hyperpolarization experiment conducted in the absence of sperm (data not shown). Peaks at the chemical shifts observed for lactate and bicarbonate were not observed in the control experiment. (**b**) Lactate, bicarbonate and pyruvate integrals versus time for washed sperm. Pyruvate integrals were divided by a factor of 10 to scale them to the same order of magnitude as the lactate integrals.

Pyruvate metabolism by live human sperm was detected through the appearance of a lactate signal at 183 ppm and a bicarbonate signal at 160.5 ppm after 150 seconds of incubation (Fig. 3a). The maximal integral for the pyruvate signal was at time zero, and its metabolism to lactate and bicarbonate resulted in their respective maximum peak integrals being observed typically after 15–20 seconds (Fig. 3b). The pH of the sperm sample was measured in the MRS tube shortly after acquiring the MRS spectra from the final hyperpolarized pyruvate addition at pH 7.3 ± 0.1 (mean ± S.D). Due to the low concentration of ^13^C_1_-pyruvic acid used in these experiments and small volume (20 μl) of neutralized pyruvate administered to the sperm sample the measured pH was a similar value to PBS buffer solution used.

Since the magnitude of metabolite signals are dependent on the number of sperm available to metabolize pyruvate, the lactate and bicarbonate response was estimated from the Area Under the Curve (AUC) (as per Fig. 3b) for washed sperm from eleven ejaculates with a concentration range of 63–573 × 10^6^/ml added to the MRS tube. Plotting the AUC ratio for lactate:pyruvate (denoted R_Lac_) against sperm concentration showed a significant correlation (r = 0.63, p = 0.04, n = 11) (Figure S1). To ascertain whether the ability of sperm to metabolize pyruvate declined with time, a second hyperpolarized pyruvate sample was added to each sperm sample approximately one hour after the first. This also showed a significant correlation with sperm concentration (r = 0.79, p = 0.02, n = 8) and analysis of covariance (ANCOVA) for R_Lac_ versus total sperm concentration linear regression found no difference in correlation between the gradient and intercept for the 1^st^ and 2^nd^ additions after controlling for sperm concentration (p = 0.39).

**Figure 4:**
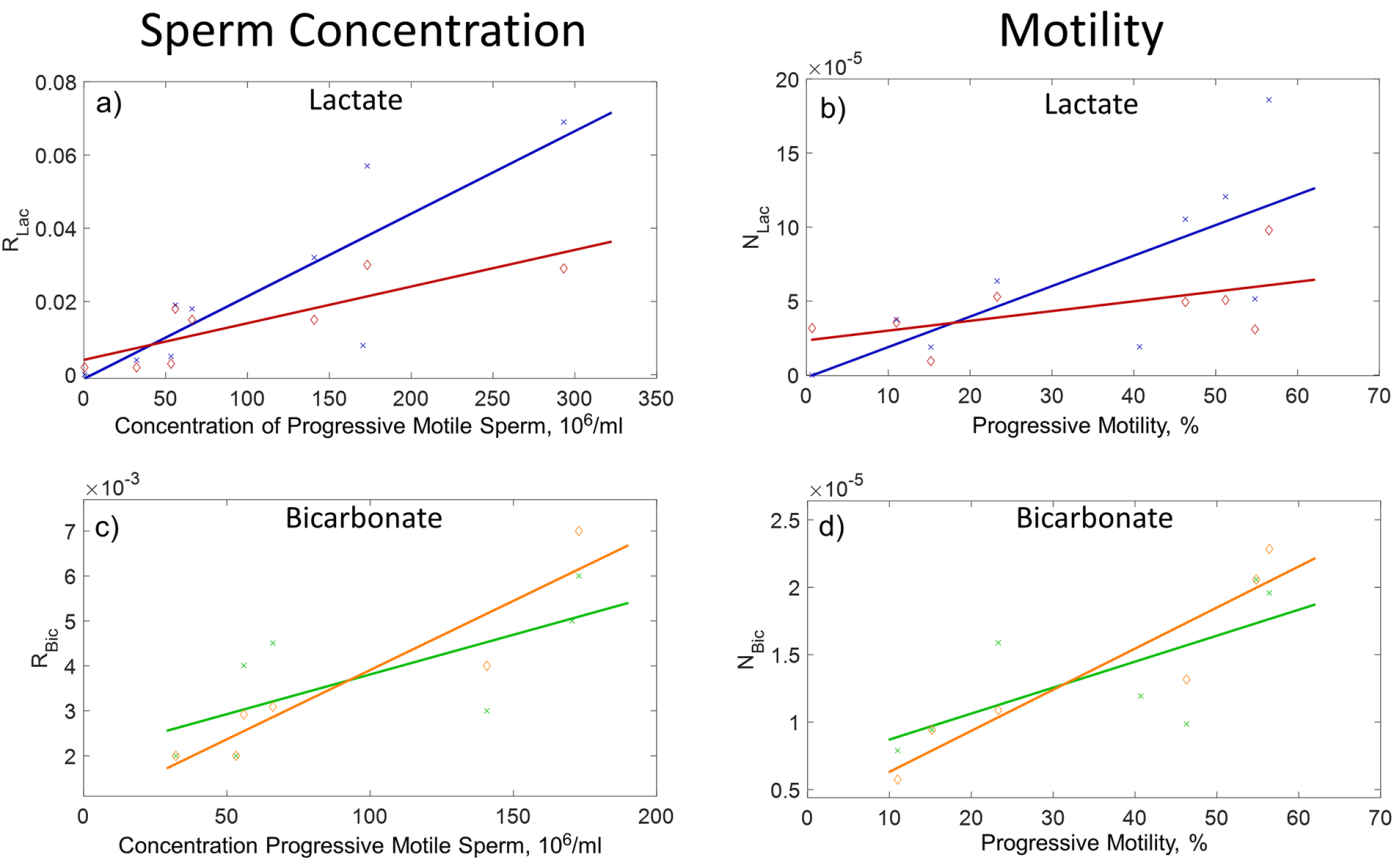
Progressive motile sperm concentration versus sperm metabolism of pyruvate to lactate, R_Lac_, (Panel a) and pyruvate to bicarbonate, R_Bic_, (Panel c). Sperm progressive motility versus total sperm concentration normalized metabolism of pyruvate to lactate, N_Lac_, (Panel b) and pyruvate to bicarbonate, N_Bic_, (Panel d). R_Lac_ and R_Bic_ were calculated as the ratio of the area under the curve for lactate:pyruvate or bicarbonate:pyruvate, respectively, from the hyperpolarized lactate, bicarbonate and pyruvate peak integrals obtained from each spectrum in the time course (180 spectra, 3 minutes). Concentration normalized, N_Lac_ and N_Bic_, values were calculated by dividing each sample value for R_Lac_ and R_Bic_ by its respective sperm concentration (10^6^/ml). Each panel shows pyruvate metabolism for sequential hyperpolarized experiments separated by ~1 hour whilst the sperm were retained in the magnet at 37 °C. The 1^st^ pyruvate addition are represented by cross markers and 2^nd^ pyruvate addition diamond markers. Sperm concentration and progressive motility were measured prior to addition to the NMR tube.

In a typical human washed sperm sample the number of progressively motile sperm represent a smaller subset of the total number of sperm. A wide range of progressive sperm motility was observed (0.7–56.5%), therefore when we calculated the concentration of progressively motile sperm and plotted this against R_Lac_ the correlation improved for both the 1^st^ pyruvate addition (r = 0.85, p = 0.004, n = 9) and 2^nd^ pyruvate addition (r = 0.84, p = 0.009, n = 8) (Fig. 4a). An ANCOVA for R_Lac_ versus progressive motile sperm concentration linear regression found no difference between the gradient and intercept for the 1^st^ and 2^nd^ pyruvate additions after controlling for progressively motile sperm motility (p = 0.06).

To determine whether there was a relationship between pyruvate to lactate metabolism and the percentage motility of sperm, the effect of sperm concentration was accounted for by normalizing each individual R_Lac_ by its respective total sperm concentration (denoted N_Lac_). Plotting N_Lac_ from the 1^st^ pyruvate addition against percent motility showed a significant positive correlation (r = 0.72, p = 0.03, n = 9) (Fig. 4b), however, a similar plot for N_Lac_ from the 2^nd^ pyruvate addition was not significant when plotted against initial sperm motility (r = 0.57, p = 0.14, n = 8) or for recovered sperm motility (r = 0.34, p = 0.41, n = 8. Data not shown) that was determined a few minutes after the experiment. An ANCOVA for N_Lac_ versus initial motility linear regression found no difference between the gradient and intercept for the 1^st^ and 2^nd^ pyruvate additions after controlling for sperm concentration (p = 0.13).

The hyperpolarized bicarbonate signal was smaller than the lactate signal with the mean AUC ratio for bicarbonate:pyruvate (denoted R_Bic_), being 3–4 times lower than the equivalent for lactate. However, it was observable in nine of the eleven washed samples examined. In contrast to R_Lac_, there was no significant correlation between R_Bic_ and sperm concentration for either the 1^st^ or 2^nd^ pyruvate addition (Figure S2). When only the concentration of progressively motile sperm was considered (Fig. 4c), there was a significant correlation with R_Bic_, for the 2^nd^ pyruvate additions (r = 0.93, p = 0.008, n = 6) but not for the 1^st^ pyruvate addition (r = 0.70, p = 0.08, n = 7). An ANCOVA for R_Bic_ versus progressively motile sperm concentration linear regression found no difference between the gradient and intercept for the 1^st^ and 2^nd^ pyruvate incubations after controlling for sperm concentration (p = 0.26).

Percentage sperm motility showed a positive correlation with metabolism of pyruvate to bicarbonate (N_Bic_) (defined as the total sperm concentration normalized values of R_Bic_). The 2^nd^ pyruvate addition bicarbonate ratio, N_Bic_ was significantly correlated with initial sperm motility (r = 0.94, p = 0.006, n = 6, Fig. 4d) and recovered sperm motility (r = 0.85, p = 0.03, n = 6. Data not shown). However, N_Bic_ versus motility for the 1^st^ pyruvate was not significant (r = 0.71, p = 0.07, n = 7, Fig. 4d), An ANCOVA for N_Bic_ versus initial motility linear regression found no difference between the gradient and intercept for the 1^st^ and 2^nd^ pyruvate additions after controlling for sperm motility (p = 0.32).

## Discussion

The data presented here show that sperm can rapidly transport pyruvate across their cellular membrane and convert it to lactate. Lactate dehydrogenase (LDH-C), is the principal LDH isoform present in sperm^26^ and the concentration of pyruvate we used in these experiments, 0.9 mM, was within the range reported as being the optimal for the conversion of pyruvate to lactate by LDH-C^27, 28^, therefore concentrations used were unlikely to overly saturate the LDH-C enzyme. Seminal plasma concentration of pyruvate is much higher than this varying between 1 and 6 mM (pp 319, Mann and Lutwark-Mann)^29^, whereas the endogenous concentration of pyruvate within the Fallopian tubes and uterus is dependent on the ovarian cycle, and is typically in the range of 0.1–0.2 mM^30, 31^. Given the observed intensity of the hyperpolarized pyruvate signal acquired, there is the possibility of further reducing the administered pyruvate concentration by an order of magnitude without adversely affecting the signal of downstream metabolites. In fact, it would be desirable to reduce the pyruvate signal so that the integrals for pyruvate, lactate and bicarbonate are within an order of magnitude of each other. This means that our experiments can be conducted within a range of physiological pyruvate concentrations, 0.1–6 mM, that sperm could expect to encounter during their journey from the cervix to Fallopian tube. Additionally, reducing the pyruvate concentration would minimize the impact of ^13^C_1_-pyruvate sample impurities on the acquired spectrum without the need to purify beyond the supplied standard.

The role of substrates within semen are to support sperm function in the early stages after ejaculation^32, 33^. Therefore, it is important to consider the influence of adding exogenous substrates, such as pyruvate, on sperm physiology. A recent study has shown that human sperm incubated for 30 minutes with 5 mM glucose and 64 nM to 5 mM pyruvate increased progressive motility by up to 21%, with an EC_50_ of 18 ± 5 μM^18^. Our study found a strong correlation between pyruvate to lactate metabolism and the degree of progressive motility when measured over a much shorter timescale of 3 minutes. However, this was only found for the 1^st^ pyruvate addition and there was no significant correlation between N_Lac_ and motility for the 2^nd^ addition. Given that there was no significant difference in the pyruvate to lactate metabolism between the two pyruvate additions the finding that N_Lac_ of the 2^nd^ pyruvate addition does not correlate with motility is unlikely to be due to sperm death.

Recently it has been shown that lactate has an inhibitory effect on sperm motility, in part by acidification of the cytosol^34, 35^, but also it has been suggested that lactate may inhibit the binding site of lactate dehydrogenase^34^. It is possible that export of lactate from sperm leads to acidification of the extracellular medium and contributes to a reduction in sperm motility. In our experiments the administered pyruvate concentration was constant, irrespective of the total number of sperm, and the extent to which pyruvate influences or is a probe of motility over this time scale warrants further investigation.

In our experiments, we observed the formation of bicarbonate from ^13^C_1_-pyruvate. Whilst bicarbonate can form via differing metabolic pathways, C_1_ labelling of pyruvate is indicative of mitochondrial activity through pyruvate decarboxylation^36, 37^. Lack of correlation between total sperm concentration and bicarbonate signal was most likely due to lower signal for bicarbonate, being 3–4 times smaller than that for lactate, and, consequently, the bicarbonate signal was more susceptible to noisy baseline variations. When only the concentration of progressively motile sperm (opposed to total sperm concentration) was used then this did significantly correlate with R_Bic_, for the 2^nd^ pyruvate injection, although not the first, suggesting a link with mitochondrial activity and motility. A similar finding for the 1^st^ and 2^nd^ pyruvate injection was found for the relationship between bicarbonate production and motility when R_Bic_ was normalized to sperm concentration and plotted against progressive motility. It has long been known that bicarbonate in seminal plasma mediates the production of cyclic adenosine monophosphate by adenylyl cyclase^38, 39^ and triggers sperm motility^39, 40^ and it is possible that detection of pyruvate to bicarbonate metabolism is a more subtle effect dependent on both sperm concentration and motility. The data showed significant correlations for lactate versus motility for the 1^st^ pyruvate injection and bicarbonate versus motility for the 2^nd^ pyruvate injection which may indicate a change from lactate fermentation to OxPhos over time. Alternatively, the initial addition of pyruvate may influence the metabolism of sperm by the time the second pyruvate addition is made, changing the preferred metabolic pathway. Combined with the effects of lactate it is possible that bicarbonate and lactate concentration may represent a feedback loop controlling overall motility^34, 35, 41^.

Measurement of lactate and bicarbonate signals would be improved by reducing the time between pyruvate sample dissolution and transfer to the sperm, mitigating hyperpolarized signal decay. Additionally, only half of the potential metabolic turnover of pyruvate was detected as the probe detection volume (200 μl) was approximately half the sample volume (~400 μl). Restricting the sperm to the detection volume and using frequency selective excitation profiles to efficiently utilize the hyperpolarized signal would provide further enhancement. Increased hyperpolarized signal would particularly improve the characterization of bicarbonate as its lower intensity, compared to lactate, means it is subject to greater noise variation. Furthermore, enhancing the overall sensitivity of the experiment would permit metabolic assessment of lower sperm counts, such as oligiozoospermic ejaculates, defined as those less than 15 million sperm per ml^2^.

In non-cancerous cells the preference for lactate fermentation or OxPhos depends upon the availability of oxygen. Within the female reproductive tract oxygen levels vary considerably for both location: 15–35 mmHg in the cervix^42^ to 6.4–32 mmHg within the uterus^43^; and patient and ovarian cycle^43^. We did not control for oxygen content of the sample which was assumed to be at atmospheric saturation levels. This could have influenced the lactate and bicarbonate signal response observed from hyperpolarized pyruvate and further work would be needed to elucidate this effect.

Although sperm rely on energy metabolism to maintain viability, not all viable sperm are motile. WHO guidelines state that sperm samples will often have a lower percentage of motile sperm than viable sperm and the interquartile range (IQR) for viable sperm in fertile men is 72–84%, whereas the IQR for motility is 47–62%^2^. The literature is unclear as to the exact relationship of lactate fermentation and OxPhos to motility and previous studies have tried to determine this by selective inhibition of one of these pathways^4^, however, this has proved inconclusive due to variation in the choice of species and experimental conditions^44^. It is likely that sperm utilize both glycolysis and OxPhos to maintain viability and motility depending on the local conditions and investigating these using selective inhibition of glycolysis and OxPhos will be conducted in future work. An alternative approach to measuring of overall sperm motility could be to correlate sperm linear velocity with the rate of lactate and bicarbonate formation from hyperpolarized pyruvate.

Dissolution DNP can be applied to many other metabolically active substrates (e.g. glucose)^25, 45^ and careful choice of ^13^C labelling strategy can allow different metabolic pathways to be examined. The advantage of the method in this paper is that these substrates can be administered sequentially or simultaneously to live sperm to highlight multiple pathways. Furthermore, this could be combined with multiple measurements of sperm metabolism at different stages of their life, such as during capacitation and hyperactivation. Discovering which substrates and sources sperm use on the way to the ovum would be important for enhancing knowledge of reproductive biology and potentially provide ideas for new treatments or solutions for men with poor sperm motility.

## Conclusion

Hyperpolarized ^13^C MRS provides a means to an understanding of how metabolic processes in sperm vary depending upon both acute changes in the local environmental conditions and during their different functional requirements. As sperm remain viable throughout the experiment additional single or multiple substrates could be used and the metabolic fluxes tracked with time, providing a ‘snapshot’ of sperm metabolism. Analysis of semen to examine fertility has changed little since being developed over 60 years ago^46^ and is limited to examining semen samples immediately after production. Studies of sperm metabolism, has the potential assess male fertility beyond the current laboratory tests and in a non-destructive manner. Such insights may also have implications for techniques of assisted reproduction or treatments to improve sperm quality.

## Methods

### Sperm preparation

All protocols used in this study involving human volunteers were approved by the University of Sheffield Research Ethics Committee (Ref No. SMBRER293. Approved 28.02.14) and were carried out according to their regulations and guidelines. Sperm samples were obtained from healthy volunteers after informed consent, with all donor information anonymized. Semen and sperm assays were performed to established protocols and WHO guidelines^2^.

After liquefaction, semen was assessed for volume, sperm concentration and motility, as outlined below. Sperm were separated from seminal fluid by carefully layering semen on top of a 40% (v/v) Percoll/PBS isotonic solution (GE Healthcare Life Sciences, Little Chalfont, UK) in a 13 ml ventilation cap tube (Sarstedt Ltd., Leicester, UK). The sample was centrifuged at 300 *g* for 20 minutes, after which the supernatant was aspirated without disturbing the sperm pellet at the bottom of the tube. PBS was then added to the sperm pellet to increase its volume by at least three times after which the sperm suspension was re-suspend and centrifuged again at 500 *g* for 10 minutes. Finally, the supernatant was aspirated and the sperm pellet was re-suspended in ~500 μl PBS. A 400 μl aliquot of washed sperm was added to a 5 mm NMR tube with 20 μl D_2_O for immediate MRS scanning.

### Semen analysis

Semen volume was determined by mass according to WHO guidelines^2^ and a Computer-Aided Sperm Analysis (CASA) system SCA version 6.1 (Microptic SL, Barcelona, Spain), Microtec LM-2 microscope (Mazurek Optical Services Ltd, Southam, UK) was used to measure sperm concentration and motility of semen and washed sperm samples. Briefly, each sample was loaded into a 10 μm 4 chamber Leja slides (Leja, Nieuw Vennep, Netherlands) and at least 200 sperm were observed across 4 to 6 microscope fields. The motility and concentration of washed sperm was measured just prior to adding the sperm to the NMR tube and again for sperm sample recovered from the NMR magnet shortly after the final hyperpolarization experiment had been completed.

### Hyperpolarization

^13^C labelled pyruvic acid (^13^C_1_-pyruvic acid (PA), Sigma Aldrich, UK) was prepared for hyperpolarization experiments by mixing, stable trityl radical OXO63 (Oxford Instruments, Abingdon, UK) at a concentration 15 mM. DOTAREM (Guerbet, Roissy, France) was added to give a final concentration of ~1.5 mM. Approximately 5.0 ± 0.1 mg (mean ± S.D.) of ^13^C_1_-labelled pyruvic acid was hyperpolarized using a HyperSense dissolution DNP polarizer (Oxford Instruments) and polarized up to >90% of maximum polarization (>40 minutes). The hyperpolarized sample was dissolved with superheated 40 mM HEPES buffer solution and transferred to an open vessel in 6 seconds. Pyruvic acid was neutralized on dissolution to its salt with a predetermined aliquot of 2.0 M NaOH solution that was previously added to the open vessel. The final injectate concentration of PA was ~19 mM. 20 μl of hyperpolarized buffered ^13^C-pyruvate solution was added to the sperm sample and well mixed to yield a final pyruvate concentration of ~0.9 mM. The time from sample dissolution to spectrum acquisition was 20–30 seconds.

### MRS Experiment

All experiments were performed on a 9.4T Bruker Avance III NMR spectrometer (Bruker BioSpin GmbH, Karlsruhe, Germany), with 5 mm broadband observe probe at 37 °C.^13^C{1H} inverse-gated spectra were acquired every 1 second for 3 minutes (Time Domain Points = 38458, Flip Angle = 16°, Sweep Width = 239 ppm, Number of Averages = 1, Repetition Time = 1 s, Number of Repeats = 180). In a subset of experiments the sperm sample was retained in the magnet at 37 °C whilst a further sample of ^13^C_1_-pyruvate was hyperpolarized. The experiment was repeated as above on the same sperm sample.

### Data and statistical analysis

Raw time domain data was processed using a custom Matlab script (Mathworks, Natick, MA, USA) to yield Fourier transformed, phase and baseline corrected spectra with 2 Hz exponential line broadening applied. Each spectrum within the time course was integrated at the chemical shift locations for pyruvate, lactate and bicarbonate peaks to create a time course plot for each metabolite. The ratio of the area under the time course curve (AUC) for lactate:pyruvate and bicarbonate:pyruvate provided an estimate of the rate of sperm metabolism for glycolysis and OxPhos respectively^47^. Further data analysis for correlation plots and statistical analysis were also made using Matlab. Assessment of statistical differences between samples used an Analysis of Covariance test (ANCOVA), Wilcoxon, two-tailed, non-parametric test and a Wilcoxon, two-tailed, matched-pairs signed rank test. Significance for both tests was p < 0.05. Pearson’s method was used for correlation plots.

### Data availability

The data that support the findings of this study are available from the corresponding author upon reasonable request.

## Electronic supplementary material

Supporting video V1

Supporting information
