# Supplementary material for: Evidence for Rapid Oxidative Phosphorylation and Lactate Fermentation in Motile Human Sperm by Hyperpolarized 13C Magnetic Resonance Spectroscopy
Source: Sci Rep. 2017 Jun 28;7:4322. doi: 10.1038/s41598-017-04146-1 (PMC5489489; doi:10.1038/s41598-017-04146-1)
Supplement: Supplementary file 2 — Supporting information [file 41598_2017_4146_MOESM2_ESM.pdf]

Evidence for Rapid Oxidative Phosphorylation and Lactate Fermentation in Motile Human Sperm by Hyperpolarized ^{13}C Magnetic Resonance Spectroscopy

Steven Reynolds[1*], Nurul Fadhlina bt Ismail[1], Sarah J. Calvert[2], Allan A. Pacey[2], Martyn N. J. Paley[1]

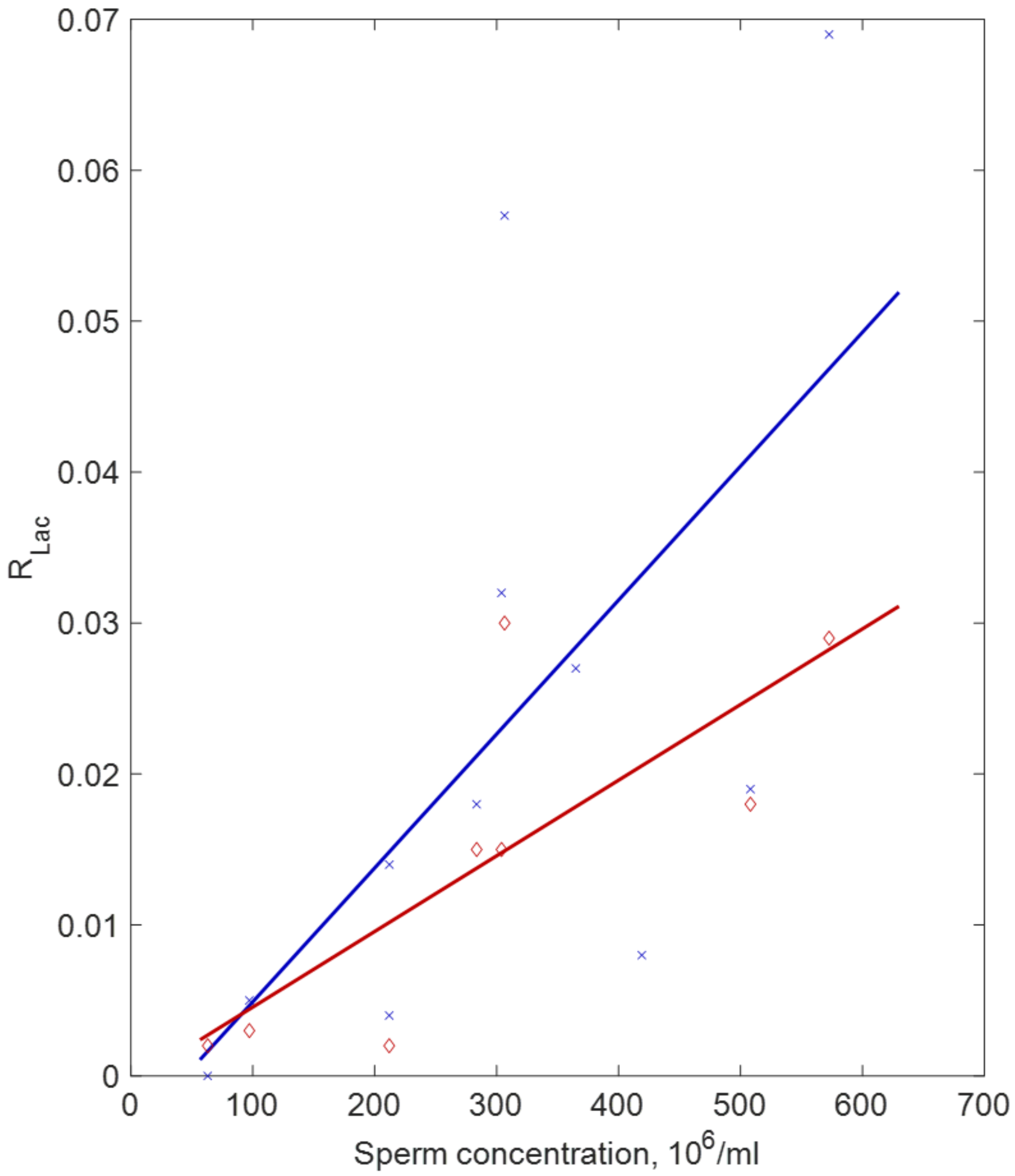

Figure S1: Ratio of Area Under the Curve (AUC) for lactate and pyruvate, R_{Lac}, versus sperm concentration measured prior to the sample being placed in the NMR tube. The AUC was calculated for both hyperpolarized lactate and pyruvate peak integrals from each spectrum in the time course (180 spectra, 3 minutes. The 1st pyruvate (crosses) and 2nd pyruvate (diamonds) additions are for sequential hyperpolarized experiments whilst retaining the sperm in the magnet at 37°C. 1st pyruvate addition, $r = 0.63$, $p = 0.04$, $n = 11$; 2nd pyruvate addition, $r = 0.79$, $p = 0.02$, $n = 8$.

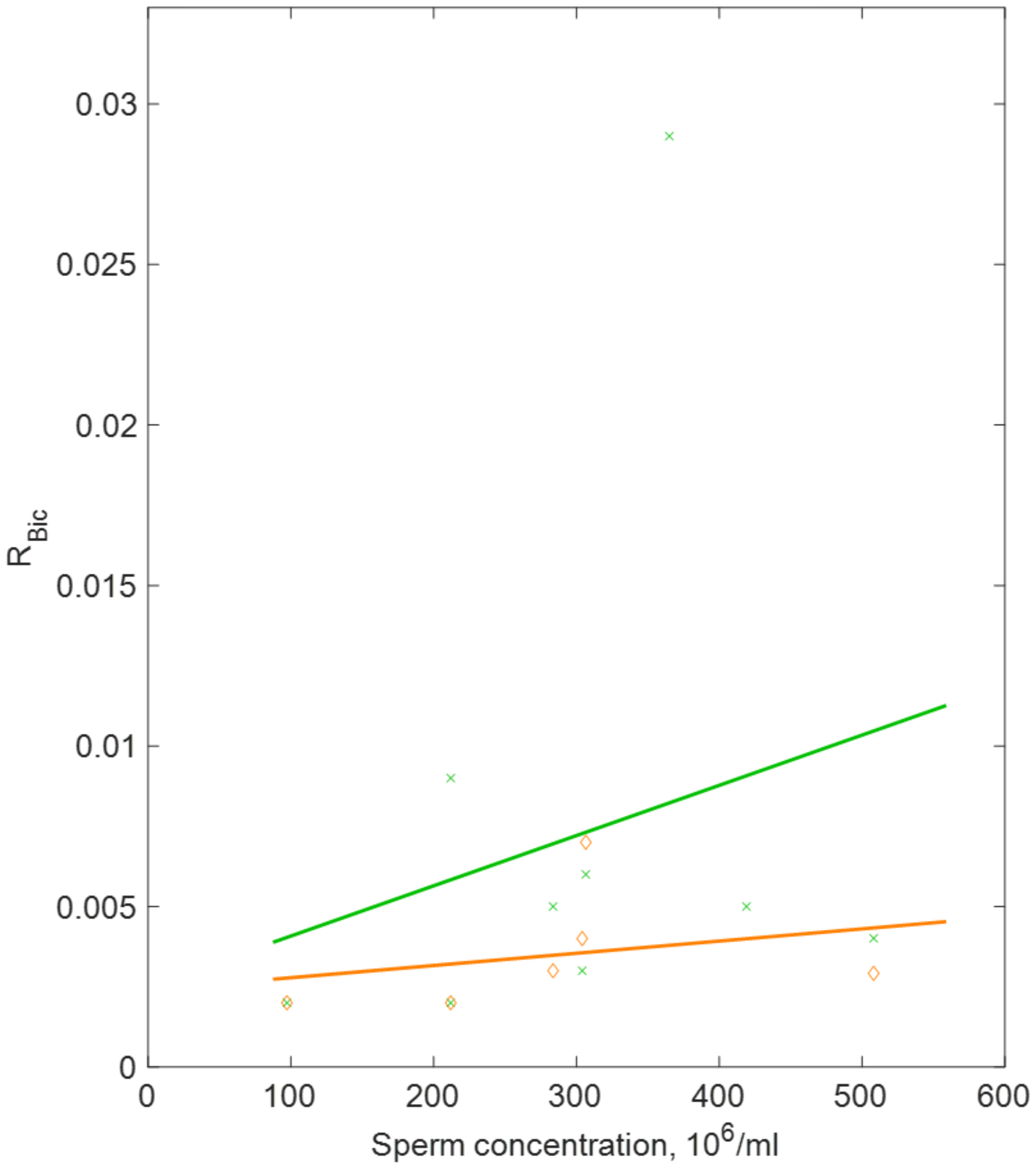

Figure S2: Ratio of Area Under the Curve (AUC) for bicarbonate and pyruvate, R_{Bic}, versus sperm concentration measured prior to the sample being placed in the NMR tube. The AUC was calculated for both hyperpolarized bicarbonate and pyruvate peak integrals from each spectrum in the time course (180 spectra, 3 minutes. The 1st pyruvate (crosses) and 2nd pyruvate (diamonds) additions are for sequential hyperpolarized experiments whilst retaining the sperm in the magnet at 37°C. 1st pyruvate addition, r = 0.23, p = 0.56, n = 8; 2nd pyruvate addition, r = 0.27, p = 0.60, n = 6.
